# Probing tissue microstructure by diffusion skewness tensor imaging

**DOI:** 10.1038/s41598-020-79748-3

**Published:** 2021-01-08

**Authors:** Lipeng Ning, Filip Szczepankiewicz, Markus Nilsson, Yogesh Rathi, Carl-Fredrik Westin

**Affiliations:** 1 Brigham and Women’s Hospital, Harvard Medical School, Boston, USA; 2 Lund University, Lund, Sweden

**Keywords:** Brain, Engineering, Biomedical engineering

## Abstract

Probing the cellular structure of in vivo biological tissue is a fundamental problem in biomedical imaging and medical science. This work introduces an approach for analyzing diffusion magnetic resonance imaging data acquired by the novel tensor-valued encoding technique for characterizing tissue microstructure. Our approach first uses a signal model to estimate the variance and skewness of the distribution of apparent diffusion tensors modeling the underlying tissue. Then several novel imaging indices, such as weighted microscopic anisotropy and microscopic skewness, are derived to characterize different ensembles of diffusion processes that are indistinguishable by existing techniques. The contributions of this work also include a theoretical proof that shows that, to estimate the skewness of a diffusion tensor distribution, the encoding protocol needs to include full-rank tensor diffusion encoding. This proof provides a guideline for the application of this technique. The properties of the proposed indices are illustrated using both synthetic data and in vivo data acquired from a human brain.

## Introduction

Noninvasive probing of cellular structure of biological tissue *in vivo* is a fundamental problem in medical science. Modern medical imaging modalities, such as computed tomography (CT) and magnetic resonance imaging (MRI), provide imaging data with spatial resolution at the scale of millimeter (mm). But mental disorders and other brain diseases are related to alternations in the cellular microstructure at the micrometer ($\upmu$m) scale without any gross effect at the macroscopic scale. However, no *in vivo* imaging technique is currently available to directly probe microscopic cellular arrangements in the human brain. Diffusion MRI (dMRI) is a modality to indirectly characterizes the microscopic cellular arrangements via the diffusion trajectories of water molecules^1,2^.

The diffusion coefficient of water at human body temperature is approximately $3\,{\upmu \hbox {m}^2\hbox {/ms}}$. In biological tissue, e.g. human brains, the displacement of water molecules is restricted or hindered by cellular membranes. The measured diffusion coefficients using dMRI, which is usually called the apparent diffusivity, depend on the microstructure of tissue as well as the experimental parameters such as the diffusion time. Within the typical 10 to 100 ms diffusion time used in dMRI, the radius of the diffusion trajectory of a water molecule is usually in the order of several $\upmu$m, similar to the size of cells. Because of microstructural heterogeneity, water molecules from different tissue components within one voxel in dMRI have different apparent diffusivity. Moreover, the diffusion trajectories can be characterized by different principal diffusion directions and different degrees of anisotropy. Thus, characterizing the statistical properties of all diffusion trajectories provide information to indirectly assess the property of tissue microstructure. Furthermore, dMRI can be performed with various diffusion encoding waveforms to sensitize the MR signal to different properties of diffusion trajectories. Then, tissue microstructure can be estimated by using a suitable analysis method for dMRI data. For example, the apparent diffusion tensor is a standard technique to characterize orientation-dependent diffusion which is related to the underlying cellular or axonal directions^3^. But this simplistic model only reflects the diffusion tensor of the ensemble average process with no information provided about the underlying variance and other high-order moments of the distribution of diffusivity of all water molecules that are useful to characterize the tissue heterogeneity.

In fact, this limitation is not only caused by the signal model but also the acquisition protocol. Specifically, dMRI data acquired by the standard linear-encoding sequence is not sufficient to characterize the variance of the distribution of apparent diffusion tensors^4^. To overcome this limitation, more advanced diffusion encoding waveforms, such as double-diffusion encoding^5,6^, q-MAS isotropic diffusion encoding^7^, and more general q-trajectory encoding (QTE)^4,8^, have been developed to characterize the microscopic anisotropy of the underlying diffusion processes. While these techniques have been used to estimate the variance of the underlying diffusion tensor distributions, the relationship between the diffusion encoding sequences and higher-order moments, e.g. skewness, remains unclear. Currently, no method is available to use the high-order moments to analyze the heterogeneity of apparent diffusion tensors within one voxel. This work introduces an approach for modeling and analyzing dMRI data acquired using novel QTE waveforms in order to derive several novel imaging indices based on the skewness of the diffusion tensor distribution. These indices are able to distinguish different distributions of diffusion tensors that cannot be set apart by current approaches, providing new imaging measures of tissue microstructure. Moreover, this work also presents a theoretical proof that full-rank b-tensors are necessary to estimate the skewness tensor. The feasibility of the proposed approach is illustrated using an *in vivo* dataset acquired from a human brain.

## Experiment and results

### Statistical indices of diffusion tensor distributions

**Figure 1:**
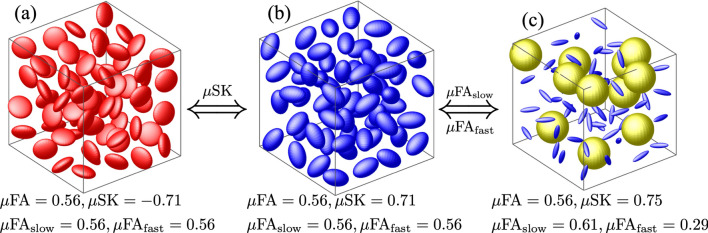
Comparison of three synthetic diffusion tensor distributions (DTDs). The yellow, red and blue-colored spheres with isotropic, oblate and prolate spherical shapes represent different tensors of different eigenvalues and orientations.

A voxel can have tissue compartments with different cellular structures that are characterized by different apparent diffusivities at long diffusion time scale. The mixture of apparent diffusivity from these tissue compartments can be represented by the diffusion tensor distribution (DTD) function, whose statistical property indirectly characterizes the tissue heterogeneity. Figure 1 compares several statistical measures of three synthetic DTDs. The eigenvalues of the diffusion tensors in $\hbox{DTD}_1$ and $\hbox{DTD}_2$ are $0.1, 0.5, 0.5\, {\upmu \hbox {m}^2\hbox {/ms}}$ and $0.634, 0.233, 0.233\, {\upmu \hbox {m}^2\hbox {/ms}}$, respectively. $\hbox{DTD}_3$ has $88\%$ of the tensors with eigenvalues being $0.63, 0.045, 0.045\, {\upmu \hbox {m}^2\hbox {/ms}}$ and $12\%$ of isotropic tensors with eigenvalues being $1.3\, {\upmu \hbox {m}^2\hbox {/ms}}$.

The three synthetic DTDs in Fig. 1 all have the same mean isotropic diffusion tensor. As a result, the three DTDs cannot be distinguished by any indices derived from the mean tensor, including the fractional anisotropy (FA) and the skewness (SK) see Methods. The microscopic anisotropy, $\upmu {{FA}}$, has been proposed to characterize more specific information of the DTDs using the underlying variances^4,7^, see Methods. But the $\upmu {\hbox{FA}}$ of the three DTDs shown in Fig. 1 have similar $\upmu {\hbox{FA}}$ values, indicating the limited sensitivity and specificity of $\upmu {\hbox{FA}}$ for characterizing tissue microstructure.

By using the third-order moment of the DTDs, we derived the microscopic skewness $\upmu {\hbox{SK}}$ and the weighted microscopic anisotropy, i.e. $\upmu {\hbox{FA}}_{\rm fast}$ and $\upmu \hbox {FA}_{\rm slow}$, to characterize the statistical property of the DTDs, see Methods. In particular, $\upmu \hbox {SK}$ is sensitive to the shape of the microscopic diffusion tensors. In Fig. 1, $\hbox{DTD}_1$ and $\hbox{DTD}_2$ have negative and positive $\upmu \hbox {SK}$ values, respectively, which reflect the underlying oblate and prolate ellipsoids for tensor representations. On the other hand, $\upmu \hbox {FA}_{\rm fast}$ and $\upmu \hbox {FA}_{\rm slow}$ are sensitive to the microscopic anisotropy of diffusion tensors with fast and slow diffusivity. $\hbox{DTD}_3$ includes isotropic tensors with relatively fast diffusivity and anisotropic tensors with relatively slow diffusivity. As a result, the underlying $\upmu \hbox {FA}_{\rm slow}$ is higher than $\upmu \hbox {FA}_{\rm fast}$. But $\upmu \hbox {FA}_{\rm fast}$ and $\upmu \hbox {FA}_{\rm slow}$ in $\hbox{DTD}_1$ and $\hbox{DTD}_2$ are not different, since the underlying tensors are of the same shape and size.

### Diffusion encoding waveforms

**Figure 2:**
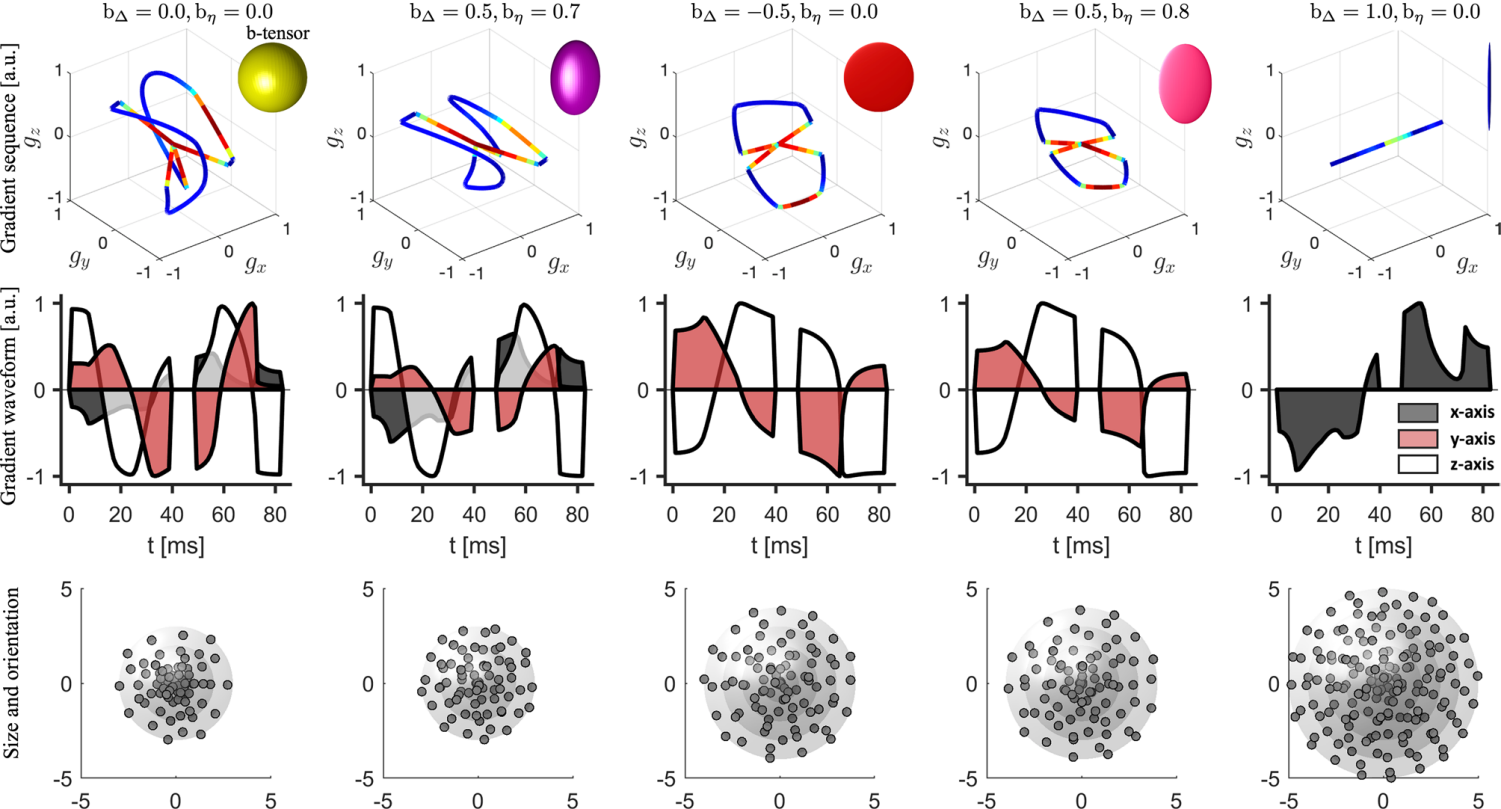
Illustration of the QTE sequences used to acquire the *in vivo* dMRI data. The plots in the first row show the 3-dimensional gradients and the shape of the corresponding b-tensors. The shape of b-tensors are determined by two parameters $b_\Delta$ and $b_\eta$ displayed on top of the plots. The curves are color coded by the slew rate, i.e. the rate of change of gradient sequences. The plots with different colors in the second row illustrate the gradient waveforms along the x-, y-, z-axises, respectively. In row three, the magnitude and orientation of a point on the spheres correspond to the trace, i.e. b-value, and an eigenvector of a b-tensor. The maximum b-values for the five types of b-tensors are equal to 3, 3, 4, 4, 5 ${\hbox{ms}}/\upmu \hbox {m}^2$, respectively. The spherical or planar shapes in the top-right corner of figures in the top row represent the “shape” of b-tensors instead of the diffusion tensors as in Fig. 1.

The proposed statistical indices have been applied to analyze an *in vivo* dataset of acquired from a human brain using a clinical 3T Siemens MAGNETOM Prisma scanner, see Methods for experimental parameters. The experiment was approved by a research ethics committee of Brigham and Women’s Hospital, Boston, MA. The first and second rows of Fig. 2 illustrate the diffusion-encoding gradient trajectories corresponding to different shapes of b-tensors, where the waveforms along x, y and z axes are coded by different colors. In the third row of Fig. 2, the rotation directions of the waveforms are shown by the dots on the spheres, and the b-values, i.e. the trace of b-tensors, are indicated by the radius of the spheres. A total number of 513 b-tensors are used in this experiment to ensure that the proposed statistical indices are uniquely determined by the dMRI signals, see Methods.

### Microstructural measures of a human brain

**Figure 3:**
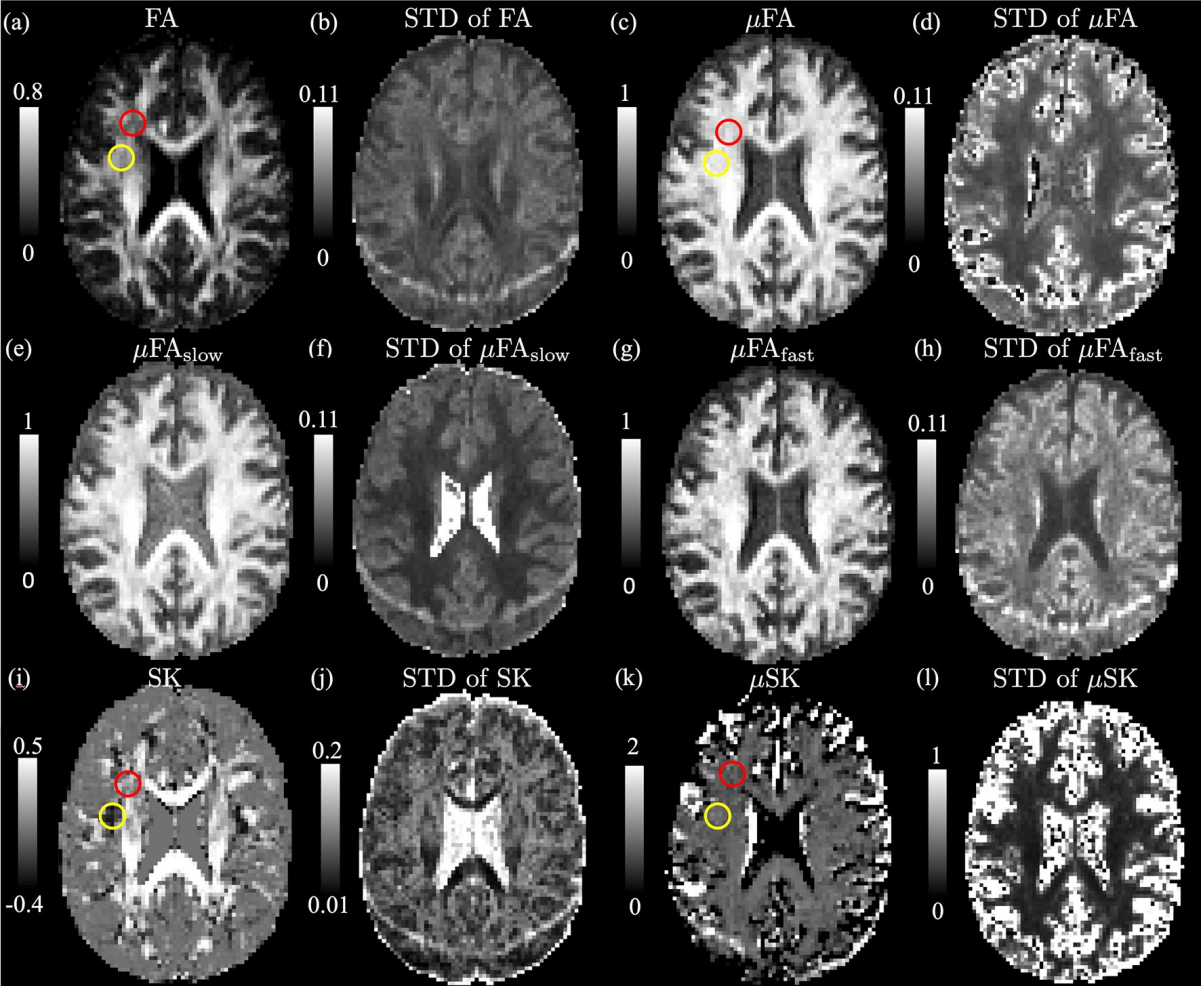
Illustration of dMRI indices and the corresponding uncertainty of an axial slice of dMRI volume from a human brain. The first row shows the $\hbox{FA}$, $\upmu \hbox{FA}$ and the corresponding standard deviation (STD). The second row illustrates the ${\upmu \hbox{FA}_{\rm slow}}$ and ${\upmu \hbox{FA}_{\rm fast}}$ measures, where ${\upmu \hbox{FA}_{\rm slow}}$ has relative higher values than ${\upmu \hbox{FA}_{\rm fast}}$. The third row shows ${{\text {SK}}}$ and ${\upmu {{\text {SK}}}}$. ${{\text {SK}}}$ has negative values in the white-matter region highlighted by the red and yellow circle whereas the corresponding $\upmu {{\text {SK}}}$ are positive, indicating that the underlying microscopic diffusion tensors have prolate shapes that are related to crossing fibers or fiber dispersion. Parameter uncertainty is inflated in a band toward the posterior of the brain. This is likely due to poor fat saturation causing a mixture of fat and water signals at this location, as previously described by^9^.

Figure 3 shows the estimated dMRI measures, including ${\hbox{FA}, \upmu \hbox{FA}, \upmu \hbox{FA}_{\rm slow}, \upmu \hbox{FA}_{\rm fast}, {{\text {SK}}}, \upmu {{\text {SK}}}}$ and the corresponding standard deviation (STD) in an axial slice of the brain. The STD values were estimated using the residual bootstrap method proposed in^10^ . The first row shows the $\hbox{FA}$, the micro-FA ($\upmu \hbox{FA}$) and their STD values. The red and yellow circles in Fig. 3a point out two white-matter regions with relative lower $\hbox{FA}$ values but with uniform $\upmu \hbox{FA}$ measures compared to other white-matter regions. The STD map in Fig. 3d shows that $\upmu \hbox{FA}$ is more reliable in white-matter. The second row shows that $\upmu \hbox{FA}_{\rm slow}$ is higher than $\upmu \hbox{FA}_{\rm fast}$ especially in gray matter. It was shown in^11^ that extra-axonal water may have slower diffusivity than the intra-axonal water. Thus, extra-axonal water may be also related to higher $\upmu \hbox{FA}$. Figure 3f,h show that $\upmu \hbox{FA}_{\rm slow}$ has lower STD values than $\upmu \hbox{FA}_\text{mfast}$ especially in white matter. The third row shows the ${{\text {SK}}}$, the micro-SK, ${\upmu {{\text {SK}}}}$ and their STD maps. The white-matter region in the yellow circle has more negative ${{\text {SK}}}$ values than the region corresponding to the red circle, though the $\hbox{FA}$ in the yellow circle is relative higher. Thus, the diffusion tensors in the yellow-circle region are more prolate and anisotropic than those from the red-circle regions. The difference in $\hbox{FA}$ and ${{\text {SK}}}$ between the two regions may be related to the number of crossing-fibers^12,13^, the fiber dispersion^14^ and/or the angle between crossing fibers^15^, which needs to be further examined. The STD of ${{\text {SK}}}$ in Fig. 3j is relatively high in white-matter, especially in regions with crossing fibers. The very high ${\upmu {{\text {SK}}}}$ values in the boundary between white/gray matter and the cerebrospinal fluid (CSF) in Fig. 3k are related to high STD values shown in Fig. 3l. It indicates that the $\upmu {{\text {SK}}}$ in these regions are sensitive to measurement noise, which may be related to the underlying partial volume effects, i.e. both CSF and white/gray matter are contained in the underlying voxel. Parameter uncertainty is inflated in a band toward the posterior of the brain as shown in Fig. 3b,f,h,j. This is likely due to poor fat saturation causing a mixture of fat and water signals at this location, as previously described by^9^.

### Accuracy and precision of diffusion indices

**Figure 4:**
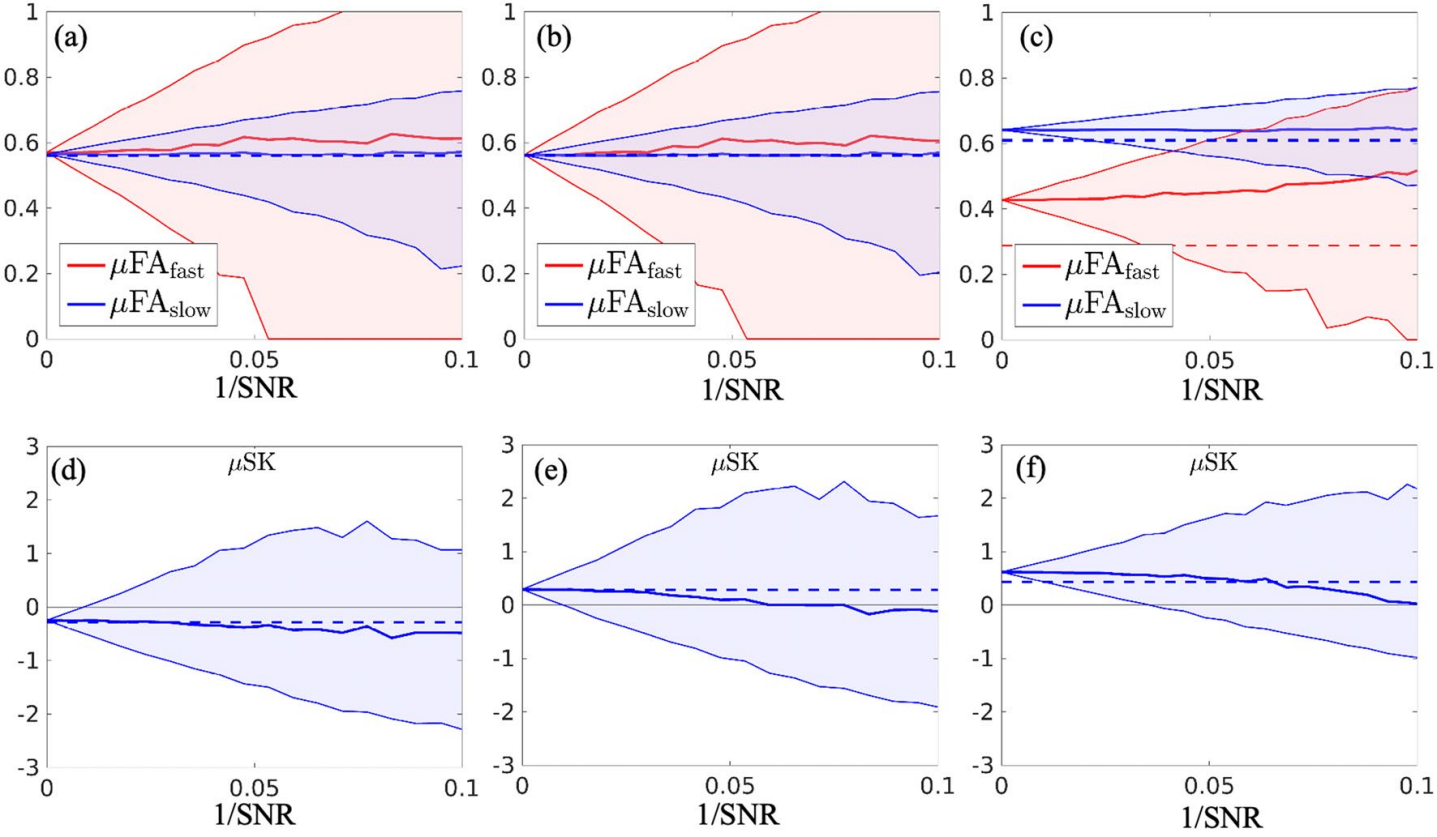
Illustration of estimated dMRI indices of the three synthetic DTDs in Fig. 1 using the diffusion encoding sequences shown in Fig. 2 with different levels of measurement noise. The horizontal axis of these plots illustrates the inverse signal-to-noise ratio (1/SNR). The upper and lower boundary of the red and blue shaded areas in (**a**)–(**c**) illustrate the 25% and 75% percentiles of the 5000 sampled values of $\upmu \hbox{FA}_{\rm fast}$ and $\upmu \hbox{FA}_{\rm slow}$ of the three synthetic structures, respectively. The red and blue solid lines show the median values of the sampled of $\upmu \hbox{FA}_{\rm fast}$ and $\upmu \hbox{FA}_{\rm slow}$ values with the underlying true values indicated by the dashed lines. Similarly, the shaded plots in (**d**)–(**f**) illustrate the 25% and 75% percentiles of the sampled $\upmu {{\text {SK}}}$ values with the median and true values shown by the solid and dashed lines.

**Figure 5:**
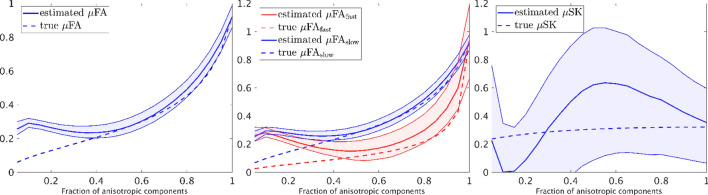
Illustration of accuracy and precision of the estimated dMRI indices of the synthetic DTDs in Fig. 1c with varying fraction of anisotropic components. The upper and lower boundary of the shaded areas illustrate the 25% and 75% percentiles of the estimated indices in simulations. The solid red or blue lines show the mean value of the estimated indices and the corresponding dashed lines show the underlying true values. The SNR of the simulated signals is equal to 30.

To examine the reliability of the estimated indices, the 513 b-tensors were applied to simulate dMRI signals for the three DTDs shown in Fig. 1 with additive Gaussian noise. The dMRI signals were computed by sampling $s(B) = \langle e^{-B:D} \rangle _\rho +\nu$ with *B* representing one of the 513 b-tensors and $\nu$ representing the zero-mean Gaussian noise. For a fixed standard deviation of Gaussian noise, the 513 dimensional dMRI signals were sampled 5000 times. Then, the same estimation methods as applied in the *in vivo* experiment were applied to estimate the $\upmu \hbox{FA}_{\rm fast}$, $\upmu \hbox{FA}_{\rm slow}$ and $\upmu {{\text {SK}}}$ measures. To further examine the accuracy and precision of the indices for different DTD functions, the experimental procedure was applied to the DTD function shown in Fig. 1c with varying volume fraction of anisotropic components and the signal to noise ratio (SNR), i.e. the ratio between the mean signal and the standard deviation of noise, being fixed at SNR=30 similar to the SNR values of the *in vivo* data.

Figure 4 illustrates the median values (solid lines) and 25% and 75% percentiles (the boarder lines of the shaded areas) of the estimated indices with different values of inverse signal-to-noise (1/SNR). The dashed lines idicate the underlying true values. The median values of $\upmu \hbox{FA}_{\rm fast}$ and $\upmu \hbox{FA}_{\rm slow}$ increase with higher noise levels. Moreover, the median values for $\upmu \hbox{FA}_{\rm fast}$, $\upmu \hbox{FA}_{\rm slow}$ in Fig. 4a,b are close to each other at different noise levels. But the median values $\upmu \hbox{FA}_{\rm slow}$ $\upmu \hbox{FA}_{\rm slow}$ in Fig. 4c are consistently far apart, indicating that the relative difference between $\upmu \hbox{FA}_{\rm fast}$ and $\upmu \hbox{FA}_{\rm slow}$ is reliable to characterize the DTDs, though these values are biased from the underlying true values. On the other hand, the median values of $\upmu {{\text {SK}}}$ in Fig. 4d–f decrease in accordance to increasing noise levels and all intersect with the underlying true values. The median values of $\upmu {{\text {SK}}}$ in Fig. 4d remain negative but the corresponding values in Fig. 4e,f gradually decrease from positive to negative values, indicating that sign of $\upmu {{\text {SK}}}$ is a reliable indices at low SNR values.

Figure 5 shows the estimated indices corresponding to the DTD shown in Fig. 1c with different volume fractions for the anisotropic components. Similar to Fig. 4, the solid red and blue lines show the median of the estimated values and the dashed lines show the underlying true values. The upper and lower boundaries of the shaded area correspond to 25% and 75% percentiles of the estimated measures. The true values stay within the shaded areas when the volume fraction of anisotropic components is higher than 0.4. But marked errors appear as the volume fraction decreases, indicating that partial volume effect may lead to biases in the diffusion indices. Moreover, the $\upmu {{\text {SK}}}$ has very high uncertainties indicating that it is challenging to be correctly estimated, which is consistent to the results of the gray matter region in Fig. 3l.

## Discussion and conclusions

This work introduces a method for estimating the variance and skewness of a distribution of diffusion tensors as an approach for characterizing the microstructure of materials or biological tissue. The method is based on expanding the diffusion tensor probability distribution in its cumulant moments, and relies on the diffusion weighting with full-rank b-tensors. The skewness of diffusion tensor^16^, diffusion skewness^17^ and high-order tensors^18–20^ have been investigated for tissue microstructure estimation based on standard (rank-one) b-tensor diffusion encoding (SDE). The added benefit of the proposed method with more general diffusion encoding is a novel capacity to distinguish diffusion tensors based on their skewness on the microscopic level, disentangling prolate and oblate shapes, as well as separately quantifying the microscopic anisotropy of diffusion tensors with high and low isotropic diffusivity. Taken together these features can add additional information on tissue microstructure, expanding on lower order methodology like DKI^21^ and QTI^4^, which have been used to characterize prostate cancer^22^ and intracranial tumors^23^. We note that the proposed model ignores the intra-compartmental non-Gaussian diffusion^24,25^ and time-dependent diffusivity^26,27^, which can be further integrated to the model to improve the accuracy for microstructure estimation. We do not expect these effects are significant in the healthy brain especially in white matter^28,29^. Moreover, the proposed indices based on cumulant moments, similar to DKI and QTI, do not directly provide specific information about tissue microstructure such as intra-axonal/cellular volume fractions, though they are sensitive to microstructural changes. From studies using cumulant expansions up to the second order we know that there exists a tradeoff between trueness and precision, where the use of higher b-values typically increase the precision while being detrimental to the trueness^30^. Nevertheless, metrics provided by such approaches can be useful in radiology for example to improve glioma grading^31^. The trueness of the parameters proposed here certainly also depends on the specifics of the imaging protocol. Further studies are needed to carefully balance the trueness and precision in this context. We emphasize the need for further investigations of tissues of pathological conditions where this may provide relevant information. We speculate that it may resolve the equality of prolate and oblate solutions and being able to robustly detect the presence of planar structures, such as those conjectured to exist in white matter sheets^15^. Currently, a proof-of-concept is illustrated in a healthy human brain *in vivo*. Indices derived from the model will be validated using liquid crystal phantoms^32^ and biological tissue in future works.

In summary, this work has introduced an approach for modeling and analyzing diffusion MRI data acquired by the novel sampling scheme with a wide range of b-tensor shapes. The main contributions include several statistical indices based on the skewness tensor of DTD functions and a mathematical proof that full-rank b-tensors are needed to uniquely determine the skewness tensor.

## Methods

### Modeling of diffusion MRI signals

In a diffusion experiment, on the application of a time-varying magnetic gradient field, ${{\varvec{g}}}(t) \in {{\mathbb {R}}}^3$ for $t\in [0, \tau ]$, the phase change of a rotating spin due to diffusive motion of water molecule during this time window is given by^33^:

$$\begin{aligned} \phi _{{\varvec{x}}}(\tau ) = \gamma \int _0^\tau {{\varvec{r}}}_{{\varvec{x}}}(t) ^T {{\varvec{g}}}(t) dt = - \int _0^\tau {{\varvec{v}}}_{{\varvec{x}}}(t) ^T {{\varvec{q}}}(t) dt, \end{aligned}$$

where $\gamma$ is the gyromagnetic ratio, ${{\varvec{r}}}_{{\varvec{x}}}(t)$ denotes the trajectory of the displacement of a particle starting from ${{\varvec{x}}}$, ${{\varvec{v}}}_{{\varvec{x}}}(t)$ denotes the corresponding velocity process, which is formally defined as ${{\varvec{v}}}_{{\varvec{x}}}(t) = {{\dot{{{\varvec{r}}}}}}_{{\varvec{x}}}(t)$, ${{\varvec{q}}}(t) = \gamma \int _0^t {{\varvec{g}}}(s) ds$ is the q-trajectory and *T* denotes the transpose. If the diffusion trajectory is assumed to be a three-dimensional Wiener process with constant diffusivity *D* during the period of diffusion time, then $\phi _{{\varvec{x}}}(\tau )$ follows a zero-mean Gaussian distribution with the covariance equal to

$$\begin{aligned} {{\mathscr {E}}}(\phi ^2_{{\varvec{x}}}(\tau )) = \gamma ^2 \int _0^\tau {{\varvec{q}}}(t)^T D {{\varvec{q}}}(t) dt = {{\text {trace}}}(BD)\triangleq B:D, \end{aligned}$$

where $B = \int _0^\tau {{\varvec{q}}}(t)^{\otimes 2} dt$ is the b-tensor. Then, the expected value of the corresponding MR signal is equal to ${{\mathscr {E}}}(e^{i\phi _{{\varvec{x}}}(T) }) = e^{- B:D}$.

The diffusion-weighted MR signal after being normalized by the non-diffusion-weighted signal, i.e. the baseline, is equal to the ensemble average of signals from all water molecules given by:

$$\begin{aligned} s(B)=\langle e^{i\phi (\tau )} \rangle _p \end{aligned}$$

where $\langle \cdot \rangle _p$ denotes the ensemble average with respect to the phase distribution function $p(\phi )$. At long diffusion time scale when the travel distances of water molecules much longer than characteristic cellular sizes, the molecular diffusion processes can be approximated by Gaussian processes with location dependent diffusivity^34^. At long diffusion time, if the mixture of apparent diffusion tensors from different cellular compartments within a voxel follows the diffusion tensor distribution (DTD) function $\rho (D)$, then the diffusion-weighted signal is equal to^4,35^:

1 $$\begin{aligned} s(B) = \int e^{-B:D} \rho (D) dD \triangleq \langle e^{-B:D} \rangle _\rho . \end{aligned}$$

A similar signal model based on scalar-valued diffusivity was investigated in^36^.

### The cumulant expansion

The standard dMRI approaches probe the diffusion process using a linear diffusion gradient waveform which corresponds to a b-tensor of rank one. In this case, estimating the underlying DTD $\rho (D)$ using an inverse Laplace transform is a highly ill-posed problem. However, advanced QTE sequences are able to probe $\rho (D)$ using any b-tensor up to rank 3, providing information that cannot be measured by SDE^5^. The ensemble average signal $\langle e^{-B:D} \rangle _\rho$ can be approximated using the following cumulant expansion:

2 $$\begin{aligned} \log s(B) \approx - B: \langle D \rangle _{\rho }+\frac{1}{2} B^{\otimes 2}: {{\mathbb {C}}}-\frac{1}{6} B^{\otimes 3}: {{\mathscr {S}}}, \end{aligned}$$

where $\langle D \rangle _{\rho }, {{\mathbb {C}}}, {{\mathscr {S}}}$ denote the first three cumulants and are given by: ${{\mathbb {C}}}= \langle (D-\langle D \rangle _{\rho })^{\otimes 2} \rangle _\rho$ and ${{\mathscr {S}}}= \langle (D-\langle D \rangle _{\rho })^{\otimes 3} \rangle _\rho$. The notation $\otimes$ denotes the tensor product and $``:''$ defines the standard inner product between matrices. For example, $B^{\otimes 3}:{{\mathscr {S}}}= [B^{\otimes 3}]_{ijk}[{{\mathscr {S}}}]^{ijk}$ using Einstein summation convention. The tensors $\langle D \rangle _{\rho }, {{\mathbb {C}}}$ and ${{\mathscr {S}}}$ have 6, 21 and 56 independent variables, respectively. These 83 variables can be estimated by solving the following linear system of equations:

3 $$\begin{aligned} \left[ \begin{array}{c} \log s_1 \\ \log s_2 \\ \vdots \\ \log s_m \end{array}\right] = \left[ \begin{array}{ccc} - \overrightarrow{B_1}'&{} \tfrac{1}{2} \overrightarrow{(B_1^{\otimes 2})}' &{} -\tfrac{1}{6} \overrightarrow{(B_1^{\otimes 3})}'\\ - \overrightarrow{B_2}'&{} \tfrac{1}{2} \overrightarrow{(B_2^{\otimes 2})}' &{} -\tfrac{1}{6} \overrightarrow{(B_2^{\otimes 3})}'\\ \vdots &{} \vdots &{} \vdots \\ - \overrightarrow{B_m}'&{} \tfrac{1}{2} \overrightarrow{(B_m^{\otimes 2})}' &{} -\tfrac{1}{6} \overrightarrow{(B_m^{\otimes 3})}' \end{array}\right] \left[ \begin{array}{c} \overrightarrow{\langle D \rangle _{\rho }} \\ \overrightarrow{{{\mathbb {C}}}} \\ \overrightarrow{{{\mathscr {S}}}} \end{array}\right] , \end{aligned}$$

where $\overrightarrow{B},\overrightarrow{\langle D \rangle _{\rho }}$ are 6-dimensional column-vector representations for *D* and *B* using the Mandel-Voigt notations such that $\overrightarrow{B}'\overrightarrow{\langle D \rangle _{\rho }}= B: \langle D \rangle _{\rho }$. Similarly, $\overrightarrow{{{\mathbb {C}}}}$ and $\overrightarrow{{{\mathscr {S}}}}$ are 21- and 56-dimensional column-vector representations for ${{\mathbb {C}}}$ and ${{\mathscr {S}}}$, respectively. Clearly, the number of measurements, *m*, needs to be at least 83 to ensure that the measurement matrix ($m\times 83$) is non-singular. The 513 b-tensors used in the experiment provides a full-rank measurement matrix of size $513\times 83$. Next we show that non-singular b-tensors are needed to ensure that the measurement matrix is of full rank.

### On full-rank b-tensors

In linear encoding sequences, the gradient sequences have varying magnitudes along a fixed direction. In this case, the corresponding b-tensors have rank one. But the corresponding 21-dimensional vector $\overrightarrow{B^{\otimes 2}}$ only spans a 15-dimensional subspace. Consequently, the variable $\overrightarrow{{{\mathbb {C}}}}$ in the linear system (3) cannot be uniquely determined using standard SDE measurements^4^. However, the vectors $\overrightarrow{B^{\otimes 2}}$ corresponding to rank-2 b-tensors given by planar encoding sequences^5,6,37,38^ or rank-3 tensors general QTE sequences^9^ are able to generate full-rank 21-dimensional space, providing a unique solution to ${{\mathbb {C}}}$. But b-tensors of rank two still have zero determinant, i.e.

$$\begin{aligned} \det (B) = b_{11}b_{22}b_{33}+2b_{12}b_{23}b_{13}-b_{13}^2b_{22}-b_{12}^2b_{33}-b_{11}b_{23}^2 =0, \end{aligned}$$

which implies that the corresponding elements in $\overrightarrow{B^{\otimes 3}}$ are linearly dependent. Therefore using only b-tensors of rank one or rank two is not sufficient to generate full-rank measurement matrices in Eq. (3). This shows that a full-rank b-tensor is needed to ensure a unique solution to Eq. (3). The importance of rank-2 b-tensors in estimating the covariance of DTDs was considered in^5,8^. The above analysis further extends the theory to show the importance of full-rank b-tensors in estimating the skewness of DTDs.

### Microscopic anisotropy and skewness

The estimated moments (or cumulants) can be used to derive scalar indices to characterize the underlying DTDs. In particular, the standard diffusion tensor imaging (DTI) technique uses the mean diffusion tensor $\langle D \rangle _\rho$ to compute several classical indices including the mean diffusivity, $\hbox{MD}= \tfrac{1}{3} {{\text {trace}}}(\langle D \rangle _\rho )$ and the fractional anisotropy $\hbox{FA}$^3^. But these measures provide limited information about the underlying DTD. For example, the mean diffusion tensor of the three DTDs illustrated in Fig. 1 is proportional to the identity matrix with $\hbox{FA}= 0,$ though the underlying DTD functions are different. One can look at the covariance tensor ${{\mathbb {C}}}$, and compute the micro-anisotropy $\upmu {\hbox{FA}}$ of the DTDs given by:

4 $$\begin{aligned} \upmu {\hbox{FA}}(\rho )^2&= \frac{3}{2} \frac{\langle \tfrac{1}{3} \sum _{i=1}^3 (\lambda _i(D) - {{\bar{\lambda }}}(D))^2\rangle _\rho }{\langle \tfrac{1}{3} \sum _{i=1}^3 \lambda _i(D)^2\rangle _\rho } \nonumber \\&= \frac{3}{2} \frac{\langle D^{\otimes 2} \rangle _{\rho } : {{\mathbb {E}}}_{{\rm shear}} }{\langle D^{\otimes 2} \rangle _{\rho } : {{\mathbb {E}}}_{{\rm iso}}}, \end{aligned}$$

where $\lambda _i(D)$ denotes the i-th eigenvalue of *D*, $\bar{\lambda }(D)=\tfrac{1}{3} \sum _{i=1}^3 \lambda _i(D)$, ${{\mathbb {E}}}_{\rm iso}= \tfrac{1}{3} I_{6\times 6}$ with $I_{6\times 6}$ being the identify matrix of size $6\times 6$ and ${{\mathbb {E}}}_{\rm shear}$ is equal to^4^

$$\begin{aligned} {{\mathbb {E}}}_{\rm shear}= \frac{1}{9} \left[ \begin{matrix}2&{} -1&{} -1&{} 0 &{}0 &{}0\\ -1&{} 2&{} -1&{} 0 &{}0 &{}0\\ -1&{} -1&{} 2&{} 0 &{}0 &{}0\\ 0&{} 0&{} 0&{} 3 &{}0 &{}0\\ 0&{} 0&{} 0&{} 0 &{}3 &{}0\\ 0&{} 0&{} 0&{} 0 &{}0 &{}3\end{matrix} \right] . \end{aligned}$$

However, in many scenarios, even the variance tensor is not enough to distinguish between the DTDs. For example, the three DTD functions in Fig. 1 have the same $\upmu {\hbox{FA}}$ measure though the underlying microscopic diffusion tensors are different. In this case, the third-order moments provide useful information to distinguish the underlying DTD functions. To this end, we propose two sets of indices, $\upmu \hbox {FA}_{\rm fast}, \upmu \hbox {FA}_{\rm slow}$ and $\upmu {\hbox{SK}}$, to characterize the DTDs using the third-order moments.

To introduce the first approach, we introduce a family of DTD functions defined by

5 $$\begin{aligned} \rho _f(D) = \frac{f(D)}{\langle f(D) \rangle _{\rho }}\rho (D), \end{aligned}$$

where *f*(*D*) is a given positive scalar-valued function of the diffusion tensor *D*. This function is chosen to scale the probability density so that the statistical property of the filtered DTD function $\rho _f(D)$ provides more specific information about the underlying distribution. The moments of the filtered DTD function $\rho _f(D)$ can be computed using:

6 $$\begin{aligned} \langle D^{\otimes k} \rangle _{f}= \int D^{\otimes k}\rho _f(D) d D = \frac{\langle D^{\otimes k} f(D) \rangle _\rho }{\langle f(D) \rangle _\rho }. \end{aligned}$$

If *f*(*D*) is a polynomial function of *D*, then both the numerator and the denominator of the above equation are linear combinations of the moments of $\rho (D)$. In particular, we consider the following two linear functions:

7 $$\begin{aligned} f_{{{\rm fast}}}(D)&= {{\text {trace}}}(D), \end{aligned}$$

8 $$\begin{aligned} f_{{{\rm slow}}}(D)&= {{\hat{d}}}-{{\text {trace}}}(D), \end{aligned}$$

where ${{\hat{d}}}$ is a positive scalar such that $f_{\hbox{slow}}(D)>0$ for all feasible diffusion tensors. The functions $f_{{\rm fast}}(D)$ and $f_{{\rm slow}}(D)$ selectively weigh the density $\rho (D)$ with relatively high and low trace values. We denote the corresponding filtered DTD function by $\rho _{{\rm fast}}$ and $\rho _{\rm slow}$, respectively. The difference between the moments of $\rho _{{\rm fast}}$ and $\rho _{\rm slow}$ reflects the distribution of $\rho (D)$ over tensors with different diffusivities.

From (6), the first and second order moments of $\rho _{{\rm fast}}(D)$ is equal to:

9 $$\begin{aligned} \langle D \rangle _{\rho _{\rm fast}}&= \frac{ \langle {{\text {trace}}}(D)D\rangle _\rho }{{{\text {trace}}}(\langle D \rangle _\rho )}, \end{aligned}$$

10 $$\begin{aligned} \langle D^{\otimes 2} \rangle _{\rho _{\rm fast}}&= \frac{\langle {{\text {trace}}}(D)D^{\otimes 2}\rangle _\rho }{{{\text {trace}}}(\langle D\rangle _\rho )}, \end{aligned}$$

which can be computed using the estimated moments of $\rho (D)$. Similarly, the first and second order moments of $\rho _{{\rm slow}}(D)$ are equal to:

11 $$\begin{aligned} \langle D \rangle _{\rho _{\rm slow}}&= \frac{ {{\hat{d}}} \langle D \rangle _\rho - \langle {{\text {trace}}}(D)D\rangle _\rho }{{{\hat{d}}} - {{\text {trace}}}(\langle D \rangle _\rho )}, \end{aligned}$$

12 $$\begin{aligned} \langle D^{\otimes 2} \rangle _{\rho _{\rm slow}}&= \frac{{{\hat{d}}} \langle D^{\otimes 2}\rangle _\rho -\langle {{\text {trace}}}(D)D^{\otimes 2}\rangle _\rho }{{{\hat{d}}} - {{\text {trace}}}(\langle D \rangle _\rho )}. \end{aligned}$$

These equations can be used to estimate specific dMRI measures using the estimated moments for $\rho _{{\rm fast}}(D)$ and $\rho _{{\rm slow}}(D)$. For the DTD functions shown in Fig. 1, the ${\upmu FA}$ measures for $\rho _{{\rm fast}}(D)$ and $\rho _{{\rm slow}}(D)$, denoted by $\upmu \hbox {FA}_{\rm fast}$ and $\upmu \hbox {FA}_{\rm slow}$, corresponding to $\hbox{DTD}_1$ and $\hbox{DTD}_2$ have the same values since the underlying diffusion tensors have the same trace. For $\hbox{DTD}_3$, $\upmu \hbox {FA}_{\rm fast}$ is much lower than $\upmu \hbox {FA}_{\rm slow}$ since the underlying fast diffusion tensors are isotropic whereas the slow-diffusion components are anisotropic.

To introduce the microscopic skewness, we first extend the skewness measure for probability distribution functions to define the following measure of asymmetry of the distribution of eigenvalues of the mean diffusion tensor $\langle D \rangle$ :

13 $$\begin{aligned} {{\text {SK}}}(\langle D \rangle )&= \frac{\tfrac{1}{3} \sum _{i=1}^3 (\lambda _i(\langle D \rangle )-{{\bar{\lambda }}} (\langle D \rangle ))^3 }{(\tfrac{1}{3} \sum _{i=1}^2 (\lambda _i (\langle D\rangle ) -{{\bar{\lambda }}} (\langle D\rangle ))^2)^{3/2}}, \end{aligned}$$

14 $$\begin{aligned}&= \frac{ \langle D\rangle ^{\otimes 3} : {{\mathscr {E}}}_{\rm shear}}{( \langle D\rangle ^{\otimes 2}:{{\mathbb {E}}}_{\rm shear})^{3/2}}. \end{aligned}$$

where $\langle D\rangle$ is assumed to be anisotropic so that the denominator is assumed nonzero. In above, ${{\mathscr {E}}}_{\rm shear}$ is a fully symmetric three-dimensional tensor, i.e. the value of $[{{\mathscr {E}}}_{\rm shear}]_{ijk}$ does not change by permuting the order of *i*, *j*, *k*, of size $6\times 6\times 6$ with the following non-zero entries

$$\begin{aligned} {[}{{\mathscr {E}}}_{\rm shear}]_{iii}&= \tfrac{2}{27} \text{ if } i \in \{1, 2, 3\},\\ {[}{{\mathscr {E}}}_{\rm shear}]_{iij}&= -\tfrac{1}{27} \text{ if } i,j \in \{1, 2, 3\} \text{ and } i\ne j,\\ {[}{{\mathscr {E}}}_{\rm shear}]_{iij}&= \tfrac{1}{18} \text{ if } i \in \{4, 5, 6\} \text{ and } j\in {{\mathfrak {I}}}_i,\\ {[}{{\mathscr {E}}}_{\rm shear}]_{iij}&= -\tfrac{1}{9} \text{ if } i \in \{4, 5, 6\} \text{ and } j\in \{1,2,3\} \backslash {{\mathfrak {I}}}_i,\\ {[}{{\mathscr {E}}}_{\rm shear}]_{ijk}&= \tfrac{\sqrt{2}}{12} \text{ if } i,j,k \in \{4, 5, 6\}, \end{aligned}$$

where ${{\mathfrak {I}}}_4=\{1, 2\}, {{\mathfrak {I}}}_5 =\{1, 3\}$, ${{\mathfrak {I}}}_6 = \{2, 3\}$. The skewness measure ${{\text {SK}}}$ is able to distinguish diffusion tensors that cannot be separated by $\hbox{FA}$ values. For example, the diffusion tensors represented by oblate tensors have a negative ${{\text {SK}}}$ while prolate tensors have positive ${{\text {SK}}}$. Following the definition of $\upmu \hbox{FA}$, we define the following microscopic skewness measure:

$$\begin{aligned} \upmu {{\text {SK}}}(\rho ) =\frac{\langle D^{\otimes 3} \rangle _\rho : {{\mathscr {E}}}_{\rm shear}}{ (\langle D^{\otimes 2} \rangle _\rho :{{\mathbb {E}}}_{\rm shear})^{3/2}}, \end{aligned}$$

which characterizes the microscopic skewness of the diffusion tensors. To enhance robustness with respect to measurement noise, the $\langle D^{\otimes 2} \rangle _\rho :{{\mathbb {E}}}_{\rm shear}$ term in the denominator on the right hand side of the above equation is replaced by $\langle D^{\otimes 2} \rangle _\rho :{{\mathbb {E}}}_{\rm shear}+ \epsilon$ with $\epsilon = 0.03\,\upmu m^4/ms^2$, which does not change the sign of $\upmu {{\text {SK}}}(\rho )$. In particular, the $\upmu {{\text {SK}}}(\rho )$ for $\hbox{DTD}_1$ in Fig. 1 is negative while for $\hbox{DTD}_2$ and $\hbox{DTD}_3$ it is positive.

### Experimental parameters of the in vivo dataset

The “free waveform” prototype pulse sequence^9^ based on a diffusion-weighted spin-echo sequence with EPI readout was used to acquire MRI data from an adult male subject. Imaging parameters were: TE = 100 ms, TR = 2800 ms, field of view = $220 \times 220 \times 75\; {\hbox{mm}}^3$, voxel size = $2.2\times 2.2\times 5\;{\hbox{mm}}^3$, partial Fourier factor = 6/8 and in-plane acceleration factor = 2. The acquired images were corrected for motion and eddy current distortion using an extrapolation-based approach capable of correcting diffusion weighted volumes acquired with high b-values^39,40^.

A total number of 513 image volumes were acquired using different QTE trajectories tailored to the MRI system by numerical optimization^41^ with compensation for concomitant gradient effects^42^. The QTE waveforms were determined by two parameters $b_\Delta$ and $b_\eta$, which characterize the shape of b-tensors^9,43^. The orientation of b-tensors was determined via rotation of the waveforms and b-values were changed by varying the magnitude of waveforms^44^. The diffusion encoding waveforms in the first two columns provide spherical and oblate b-tensors with full-rank, which are required to estimate the skewness of the underlying DTD. The 513 b-tensors in this experiment provide a full-rank measurement matrix so that the proposed statistical indices are uniquely determined by the dMRI signals.
